# Supplementary material for: Dexamethasone Downregulates Autophagy through Accelerated Turn-Over of the Ulk-1 Complex in a Trabecular Meshwork Cells Strain: Insights on Steroid-Induced Glaucoma Pathogenesis
Source: Int J Mol Sci. 2021 May 31;22(11):5891. doi: 10.3390/ijms22115891 (PMC8198647; doi:10.3390/ijms22115891)
Supplement: Supplementary file 1 [file ijms-22-05891-s001.zip › ijms-1083767-supplementary.pdf]

SUPPLEMENTARY FIGURES

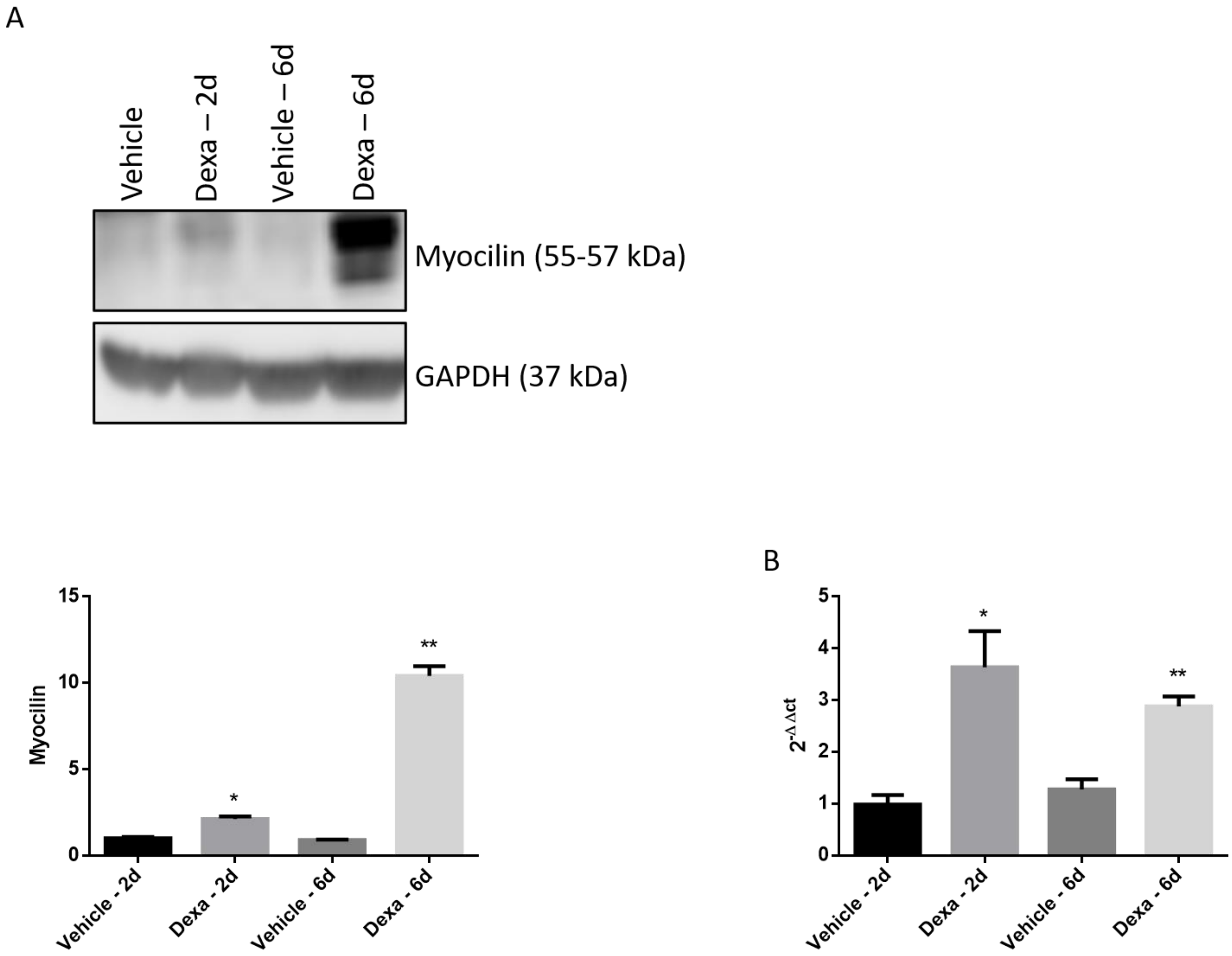

Figure 1: **Validation of TMC strain identity**. (A) Myocilin staining of Dexa-2d and Dexa-6d cells (and correspondent Vehicle cells) by denaturing and reducing Wb. GAPDH was used as internal control. A representative image of three independent experiments is here shown (n=3). One-way ANOVA followed by Tukey's post-hoc significance test. *p<0.001; **p<0.00001; (B) RT-PCR analysis of myocilin gene expression. GAPDH was used as internal control. Values were determined through the 2-ΔΔCt formula. *p<0.0001, **p<0.0015.

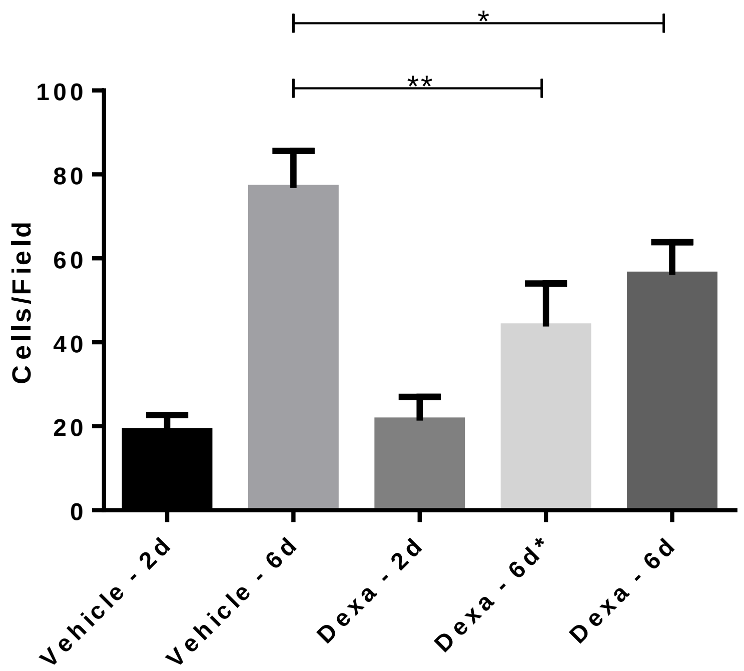

Figure 2: **TMCs cell count by Trypan blue**. Cells of each experimental condition were counted in 9 different fields upon Trypan blue staining. A representative image of two independent experiments is here shown (n=9). One-way ANOVA followed by Tukey's post-hoc significance test. *p<0.0001, **p<0.004

A

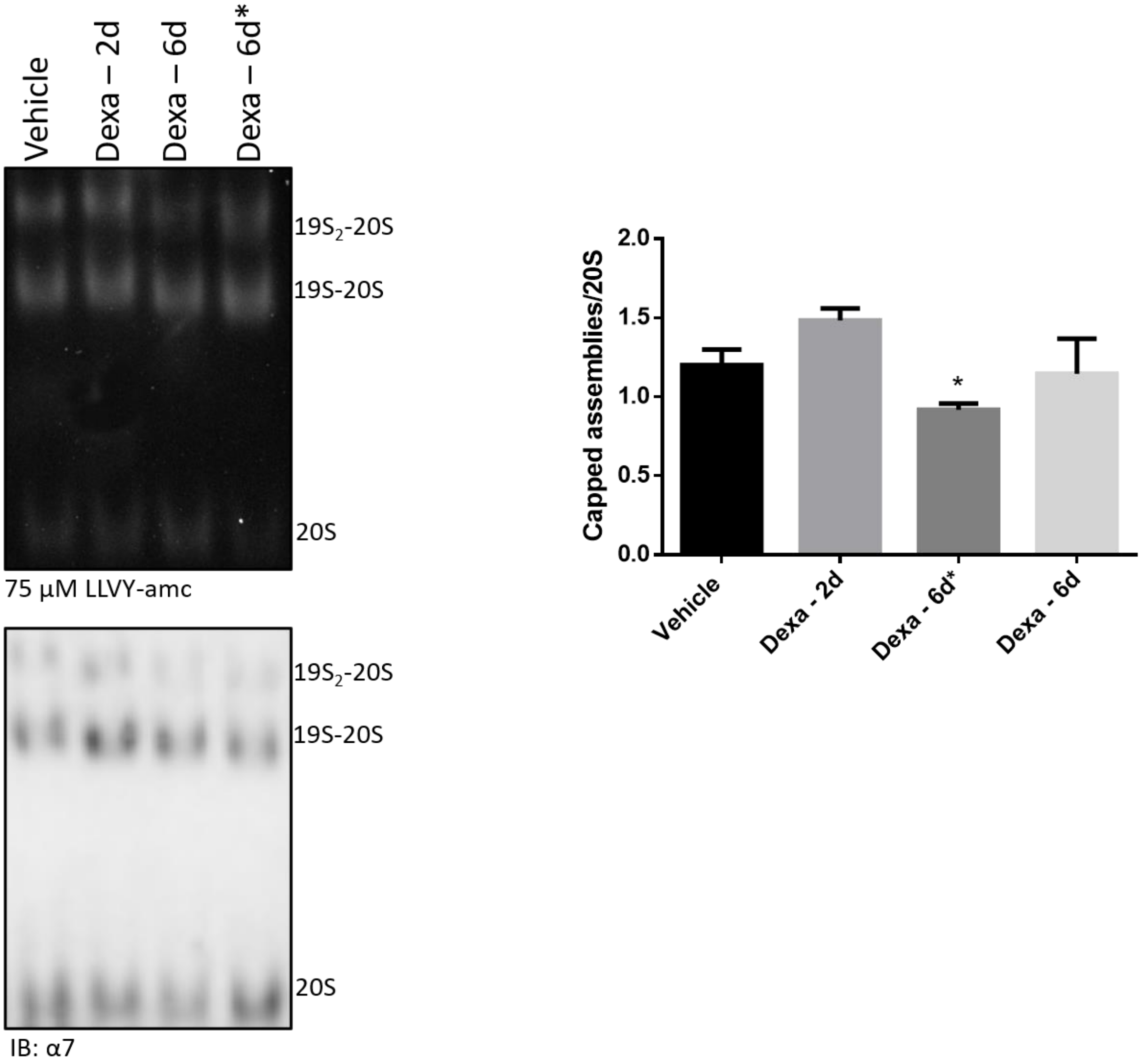
Vehicle
Dexa – 2d
Dexa – 6d
Dexa – 6d*
19S2-20S
19S-20S
20S
75 µM LLVY-amc
19S2-20S
19S-20S
20S
IB: α7
Capped assemblies/20S
2.0
1.5
1.0
0.5
0.0
*
Vehicle
Dexa - 2d
Dexa - 6d*
Dexa - 6d

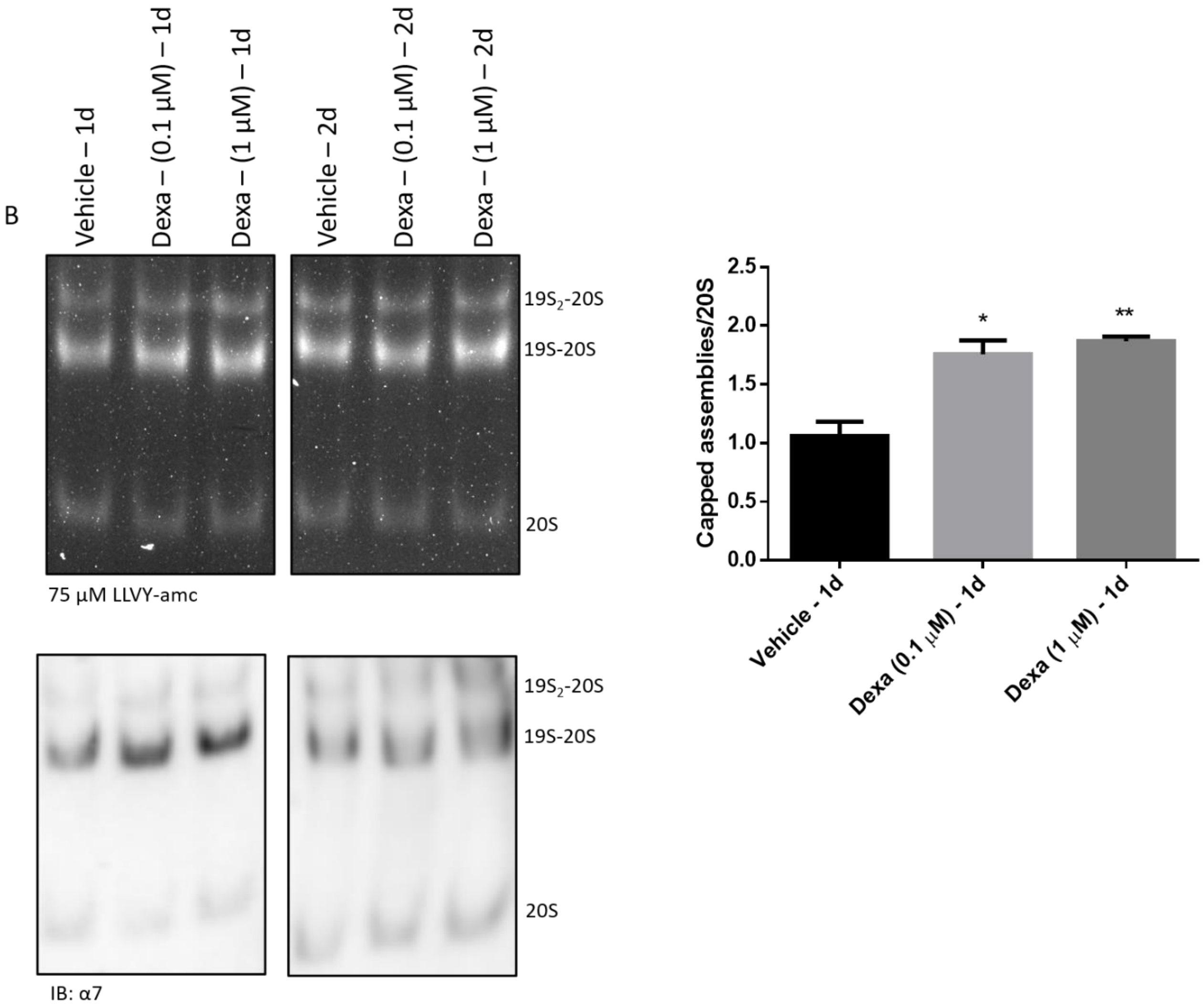

Figure 3: Assessment of proteasome activity and assembly by native-gel electrophoresis. (A) Proteasome particles were separated by mass/charge through native gel electrophoresis. The three main assemblies were probed with 75 µM LLVY-amc (*upper panel*). The identity of the particles was verified by staining them with an anti-α7 antibody (*lower panel*) *p<0.003; (B) proteasome assemblies of TMCs stimulated for 1 day and 2 days with 0.1 µM and 1 µM Dexa. *p<0.0004, **p<0.0001. In all cases quantification is reported as the sum of capped assemblies intensity vs that of 20S as they appear upon immunostaining. A representative experiment of three independent observations is reported (n=3); One-way ANOVA followed by Tukey's post-hoc significance test.

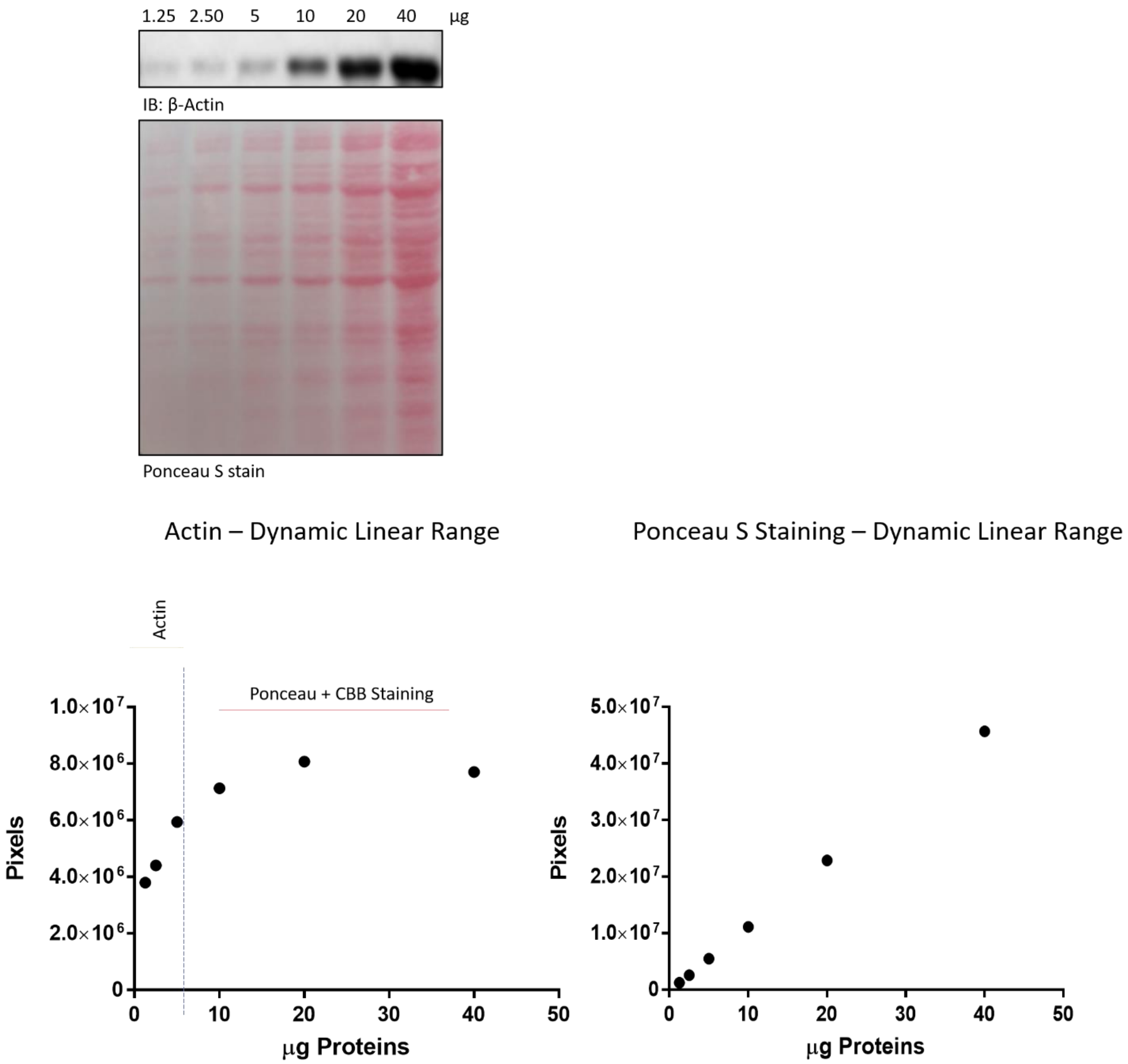

Figure 4: **Determination of β-actin and Ponceau S staining dynamic linear range**. Serial dilution (from 40 µg to 1.25 µg) of a TMCs lysate were analysed by Western blotting and probed with the anti-β-actin antibody. Bands intensity was determined by ImageJ and data plotted to visualize the range within which the signal was below the saturation threshold. Thereafter, proteins demanding µg loading higher that the saturation threshold normalized to Ponceau S staining (and confirmed by CBB, not shown).
